# Enhanced Isolation and Detection of COVID-19 in Hospitalized Patients Undergoing Antiviral Therapy

**DOI:** 10.3201/eid3201.251011

**Published:** 2026-01

**Authors:** Ranawaka A.P.M. Perera, Andrew Marques, Jevon Graham-Wooten, Li Hui Tan, Noam Cohen, Kyle Rodino, Ronald G. Collman, Frederic D. Bushman, Susan R. Weiss

**Affiliations:** Department of Veterans Affairs, Philadelphia, Pennsylvania, USA (R.A.P.M. Perera); University of Pennsylvania, Philadelphia (R.A.P.M. Perera, A. Marques, J. Graham-Wooten, L.H. Tan, N. Cohen, K. Rodino, R.G. Collman, F.D. Bushman, S.R. Weiss)

**Keywords:** SARS-CoV-2, coronavirus disease, COVID-19, qRT-PCR, sequencing, virus culture, infectivity, viruses, respiratory infections, antiviral therapy, pandemic preparedness, United States

## Abstract

We evaluated the efficiency of SARS-CoV-2 detection from patient respiratory specimens by comparing 3 cell lines: Vero E6, Vero E6 expressing transmembrane protease serine 2 (Vero E6 T2), and Vero E6 expressing angiotensin-converting enzyme 2 and transmembrane protease serine 2 (Vero E6 A2T2). We compared a range of sample types, clinical conditions, and real-time reverse transcription PCR cycle threshold values. Vero E6 A2T2 exhibited enhanced sensitivity by supporting efficient virus entry and replication with faster cytopathic effect. Vero E6 culture isolated infectious virus only up to 3 days after PCR confirmation but with Vero E6 A2T2 cells, culture occurred up to 7 days after confirmation. Whole-genome sequencing showed no evidence of adaptive mutations when Vero E6 A2T2 was used for viral culture, supporting use for downstream analyses. Optimized infectious virus detection systems are needed for research and clinical settings, particularly for high-risk, immunocompromised populations that produce virus longer and contribute to variant emergence.

After infection, SARS-CoV-2 is produced from the respiratory tract, which is the primary mode of secondary transmission between contacts (*1*–*3*). Therefore, it is essential to identify and isolate patients producing infectious virus to minimize transmission. Developing sensitive methods for identifying infectious virus and defining the kinetics of infectious viral production are critical for informing measures aimed at reducing the community and hospital transmission risk. This goal is particularly crucial for hospitalized or immunocompromised persons who might experience prolonged viral replication with consequences of both prolonged infectivity and new variant emergence, as well as receiving antiviral treatments whose effect on infectious virus produced is not well understood (*4*,*5*)

Infected persons might remain real-time reverse transcription (qRT-PCR) positive for extended periods well after the resolution of clinical symptoms and detectable infectious virus (*6*–*8*). Previous studies have associated antigen positivity with detectable infectious virus (*9*). Alternatively, some practitioners use specific qRT-PCR cycle thresholds (Cts) or defined time after initial PCR positivity (*10*–*12*). However, sensitive detection of infectious virus is essential to know which patients might contribute to forward transmission and to calibrate other methodologies.

Typically, the Vero E6 cell line has been used for the detection of infectious virus production and the resultant data was the basis for designating isolation measures during the initial pandemic phase (*13*–*15*). This cell line is particularly advantageous because of its genetic deficiency in type I interferon production, which enables efficient viral propagation by evading host antiviral defenses (*16*,*17*) However, highly sensitive methods are necessary to understand the nature of infectious virus production, particularly in hospitalized patients undergoing antiviral treatment. Subsequent studies have used modified Vero E6 lines expressing transmembrane protease serine 2 (TMPRSS2) and angiotensin-converting enzyme 2 (ACE2) for improved viral culture (*18*–*21*) but without systematic parallel comparisons across these lines. The purpose of this study was to compare viral culture methods for the detection of infectious SARS-CoV-2 to identify improved and optimized methods.

## Materials and Methods

### Patient Population

We obtained respiratory samples from 246 SARS-CoV-2 positive patients at the Hospital of the University of Pennsylvania (Philadelphia, PA, USA), determined by Cepheid (https://www.cepheid.com), Thermo Fisher TaqPath EUA (Thermo Fisher Scientific, https://www.thermofisher.com), or Roche Cobas (https://www.roche.com) clinical assay, depending on period. We determined Ct values for all samples before virus isolation by the qRT-PCR protocol as described previously (*22*). Nasopharyngeal swab and endotracheal aspirate specimens were collected during April 2020–February 2024 from critically ill hospitalized patients who provided consent (University of Pennsylvania internal review board protocol no. 823392), and we extracted clinical data from the electronic medical record system. We obtained additional nasopharyngeal swab specimens as deidentified specimens that remained after clinical patient testing from several timepoints during April 2020–February 2024 (University of Pennsylvania internal review board protocol no. 814859). Nasopharyngeal swab specimens were collected in viral transport medium (VTM; BD, https://www.bd.com) or in some cases phosphate-buffered saline (PBS) because of VTM shortage, and endotracheal aspirates were collected without media. Specimens were stored at −80C until analysis.

### Cell Lines and Viruses

We cultured Vero E6 (ATCC-CRL-1586), Vero E6 TMPRSS2 (kindly provided by Dr. Sara Cherry), and Vero E6 ACE2 TMPRSS2 (kindly provided by Dr. Luis Martinez-Sobrido) in Dulbecco minimum essential medium (DMEM) with 10% L-glutamine, 4.5 g/L D-glucose (ThermoFisher Scientific), 10% heat-inactivated fetal bovine serum (FBS; Cytiva, https://www.cytivalifesciences.com), and 1× penicillin/streptomycin (Thermo Fisher Scientific). rSARS-CoV-2-mCherry virus was kindly provided by Dr. Luis Martinez-Sobrido.

### Human Primary Epithelial Cell Air-Liquid Interface Cultures

We derived nasal epithelial stem cells from cytologic brushings obtained from patients without respiratory infection undergoing sinonasal surgery at the University of Pennsylvania and the Philadelphia Veterans Affairs Medical Center, after receiving informed consent and under protocols approved by the University of Pennsylvania internal review board (protocol no. 800614) and the Philadelphia Veterans Affairs internal review board (protocol no. 00781). We pooled nasal cells from 4–6 patients and differentiated as previously described to prepare air-liquid interface cell cultures (*23*).

### Virus Culture

We seeded Vero E6, Vero E6 TMPRSS2, and Vero E6 ACE2 TMPRSS2 cells at 100,000 cells/well into 48-well plates (TPP Techno Plastic Products, https://www.tpp.ch) to achieve subconfluent monolayers after 24 hours in a CO_2_ incubator. We removed the medium and inoculated 50 µL of PCR-positive clinical specimen diluted 1:1 in DMEM with 2% fetal calf serum (both Thermo Fisher Scientific) in triplicate. After 1 hour incubation at 37°C, we added 1 mL of 2% FBS DMEM. We incubated the plates at 37°C in a CO_2_ incubator. We observed the cells daily and harvested when 40%–50% demonstrated cytopathic effect.

### Whole-Genome Sequence Analysis

We sequenced genomes by using the ARTIC POLAR protocol as described previously (*24*,*25*). We analyzed the genomes after aligning to the SARS-CoV-2 wild-type reference sequence (GenBank accession no. NC_045512.2). We used the BWA aligner tool version 0.7.17 (https://github.com/lh3/bwa/releases/tag/v0.7.17) with a filter requiring a minimum mapping score of 30. We removed reads that did not align to the reference by using Samtools version 1.10 (https://github.com/samtools/samtools/releases/tag/1.10). We called variants by using Bcftools version 1.10.2-34 (https://github.com/samtools/bcftools/releases/tag/1.10.2). We used a previously published bioinformatics pipeline to assign point mutations (*26*–*29*).

### Infection of Nasal Air-Liquid Interface Cultures

We diluted virus isolates in 2% FBS DMEM to 50 µL at multiplicity of infection (MOI) of 0.01 and added apically to nasal air-liquid interface (ALI) cultures for 1 hour adsorption. Then, we apically washed the cells 3 times. We added 100 µL of 2% FBS DMEM apically to collect the produced virus.

### Tissue Culture Infectious Dose Assays

We used Vero E6, Vero E6 TMPRSS2, or Vero E6 ACE2 TMPRSS2 cells for 50% tissue culture infectious dose (TCID_50_) assays. We added serial dilutions onto cell plates that were incubated at 37°C for 1 hour. We added a liquid overlay (DMEM with L-Glut, 2% heat-inactivated FBS, 1% sodium pyruvate, 0.01% agarose) and incubated for 16 hours. We removed the overlay and added 4% paraformaldehyde for >30 minutes to fix monolayers. We removed the paraformaldehyde and washed 3 times in 0.05% Tween/PBS (Thermo Fisher Scientific) and blocked with 2% bovine serum albumin in PBS with Tween 20 (Thermo Fisher Scientific). Then, we added 50 µL SARS-CoV-2 nucleocapsid antibody and incubated overnight. We washed the plates 3 times, added 50 µL horseradish peroxidase-conjugated secondary antibody, incubated for 1 hour, washed the plates again, added 50 µL KPL TrueBlue substrate (SeraCare, https://www.seracare.com), and incubated for 15 minutes at room temperature. After that incubation, we washed the plates 3 times with distilled water. We estimated the endpoint titer (50% of wells positive) by using the Reed-Muench method.

### Western Blots

We rinsed cells with PBS stored on ice. We prepared lysates by using lysis buffer (1% nucleocapsid-40, 2 mM EDTA, 10% glycerol, 150 mM NaCl, 50 mM Tris-HCl, pH 8.0) with protease and phosphatase inhibitors (Roche). We conducted Western blots as previously described (*23*).

### Statistics

We graphed and analyzed data by using GraphPad Prism (https://www.graphpad.com), showing individual values or mean ±SD. Unless stated, we determined significance by Fisher exact test for pairwise comparisons.

### Biosafety

We conducted all procedures in a certified Biohazard Safety Level 3 laboratory. Procedures were approved by the University of Pennsylvania Office of Environmental Health and Safety.

## Results

Nasal swab or endotracheal aspirate specimens were collected and analyzed from a total of 246 patients infected with SARS-CoV-2. The cohort included cross-sectional samples (n = 178) and longitudinal samples (n = 68) collected from patients. Among the patients from whom longitudinal samples were collected, 10 had asymptomatic infection, 54 had symptomatic respiratory illness and survived, and 4 died (Tables 1, 2). Of those 68 patients, 23 were male and 45 were female. The median patient age was 61 (range 24–101) years. Major comorbidities and immunosuppression were noted in 25 cases. Hematologic malignancies such as leukemia and lymphoma were present in 11 patients, 5 patients had undergone solid organ transplantation, and 10 had solid malignancies on active therapy. 

**Table 1:** SARS-CoV-2 strains recovered from PCR positive patients in study on the enhanced isolation and detection of COVID-19 in hospitalized patients undergoing antiviral therapy

SARS-CoV-2 strain	PCR positive patients	
	Cross-sectional	Longitudinal
Wild-type	5	30
Delta	6	0
BA.1	12	0
BA.2	34	0
BQ	25	0
BA.5	70	0
XBB.1	26	0
Unknown	0	38
Subtotal	178	68
Total	246	

**Table 2:** Longitudinal patient cohort demographics from study on the enhanced isolation and detection of COVID-19 in hospitalized patients undergoing antiviral therapy*

Characteristics	Value
Patients	68
Age range, y	24–101
Sex	
M	23
F	45
Clinical manifestation	
Asymptomatic	10
Symptomatic, survived	54
Symptomatic, died	4
Vaccinated	38
Nonvaccinated	30
Antiviral treatment	
Remdesivir	27
Paxlovid	3
Monoclonal antibody	6
No antivirals	7
Immunosuppressed	25

*Values are no. patients except as indicated. Paxlovid, http://www.pfizer.com.

From the longitudinal cohort, 38 patients who sought care during the later stages of the pandemic were vaccinated and had received booster doses, whereas the remaining 30 patients, who sought care during the early stages of the pandemic, were unvaccinated. Of patients who received therapy, 27 received remdesivir, 3 Paxlovid (Pfizer, https://www.pfizer.com), and 6 monoclonal antibody therapy before specimen collection. Most patients also received corticosteroids as part of the treatment regime. 

**Figure 1:**
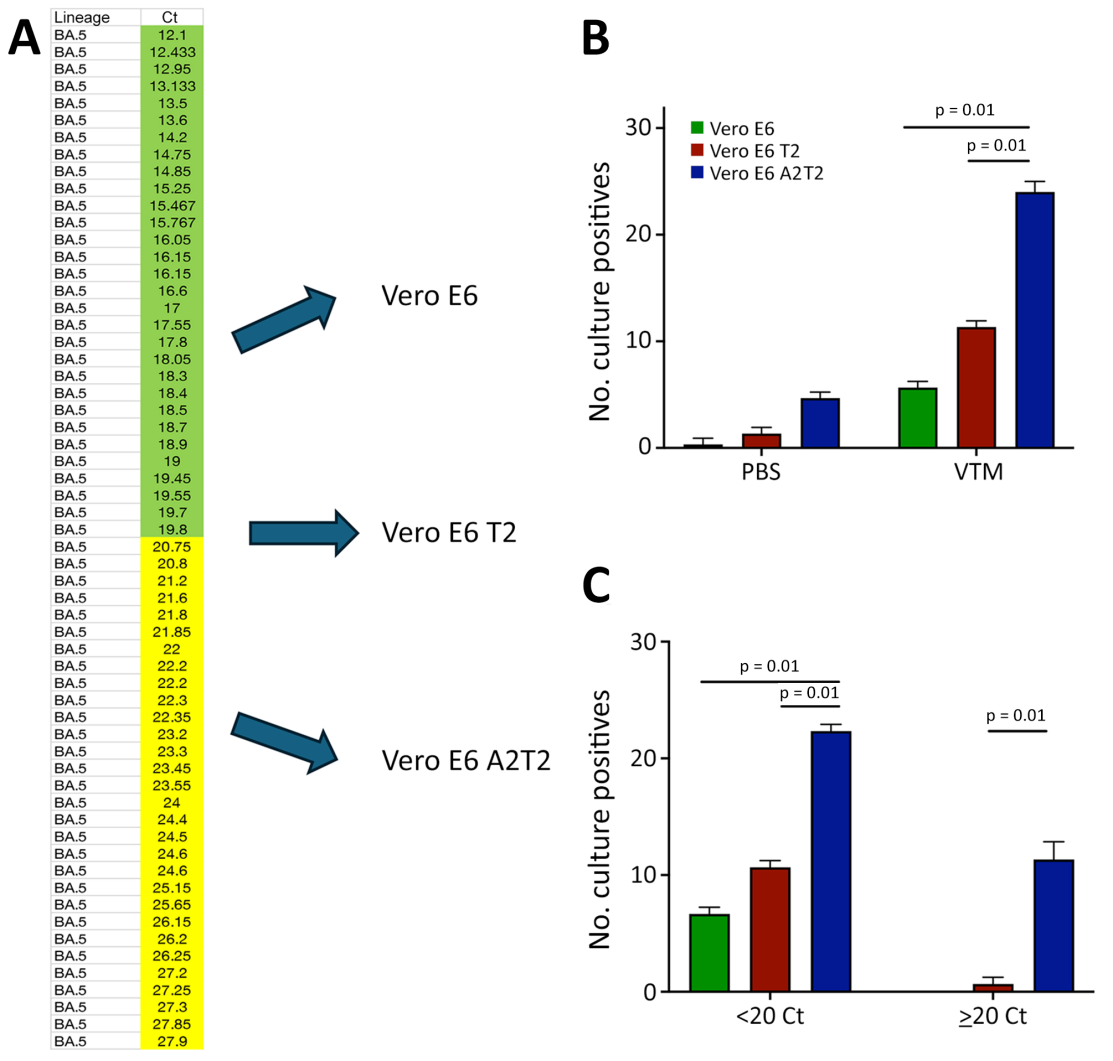
Comparative analysis of SARS-CoV-2 infectious virus isolation using Vero E6–derived cell lines from study on the enhanced isolation and detection of COVID-19 in hospitalized patients undergoing antiviral therapy. A) Thirty nasal swab specimens confirmed by real time PCR to contain the SARS-CoV-2 BA.5 variant, representing a range of Ct values from 12.1–27.9, were inoculated in triplicate onto Vero E6, Vero E6 T2, and Vero E6 A2T2 cell lines. B) Number of BA.5-positive nasal swab specimens collected in either PBS or VTM and inoculated in triplicate onto the 3 cell lines. C) BA.5-positive nasal swab specimens collected in VTM and stratified by Ct values; samples with values <20 or >20 were inoculated in triplicate into the 3 cell lines, and the number of successful virus isolations was plotted with corresponding means +SD. Error bars indicate SDs. Ct, cycle threshold; PBS, phosphate-buffered saline; Vero E6 T2, Vero E6 cells expressing transmembrane protease serine 2; Vero E6 A2T2, Vero E6 cells expressing both transmembrane protease serine 2 and angiotensin-converting enzyme 2; VTM, viral transport medium.

We carried out virus culture isolations by using the routinely used parental Vero E6 cell line; Vero E6 expressing the serine protease TMPRSS2 (Vero E6 T2), which enables SARS-CoV-2 spike protein processing and enhances viral entry; and Vero E6 expressing both TMPRSS2 and ACE2, which serves as the receptor for SARS-CoV-2 entry (Vero E6 A2T2). We evaluated those 3 cell lines in parallel for their efficiency in supporting virus culture isolation (Figure 1, panel A). 

To assess the effect of collection medium on virus isolation efficiency, we inoculated nasal swab specimens collected with PBS and VTM and confirmed to contain the BA.5 SARS-CoV-2 variant onto 3 cell lines (n = 30 per group with comparable Ct values). The virus isolation rates from PBS samples were 3.3% (n = 1) in Vero E6, 6.6% (n = 2) in Vero E6 T2, and 16.6% (n = 5) in Vero E6 A2T2. In contrast, VTM samples demonstrated markedly higher isolation efficiencies: 20% (n = 6) in Vero E6, 40% (n = 12) in Vero E6 T2, and 83.3% (n = 25) in Vero E6 A2T2 (p<0.001) (Figure 1, panel B).

To evaluate the sensitivity across a range of viral copy numbers in nasal swab specimens, we stratified samples collected only in VTM confirmed to contain BA.5 by qRT-PCR Ct values, <20 (n = 30) or >20 (n = 30), and inoculated both onto the 3 cell lines separately. When Ct values were <20, all 3 cell lines supported virus isolation, with efficiencies of 23.3% (n = 7) for Vero E6, 36.6% (n = 11) for Vero E6 T2, and 76.6% (n = 23) for Vero E6 A2T2 (p<0.001). However, for samples with Ct values >20, only Vero E6 T2 (3.3%, n = 1) and Vero E6 A2T2 (43.3%, n = 13) successfully supported viral isolation (p<0.001); Vero E6 failed to yield virus (Figure 1, panel C).

**Figure 2:**
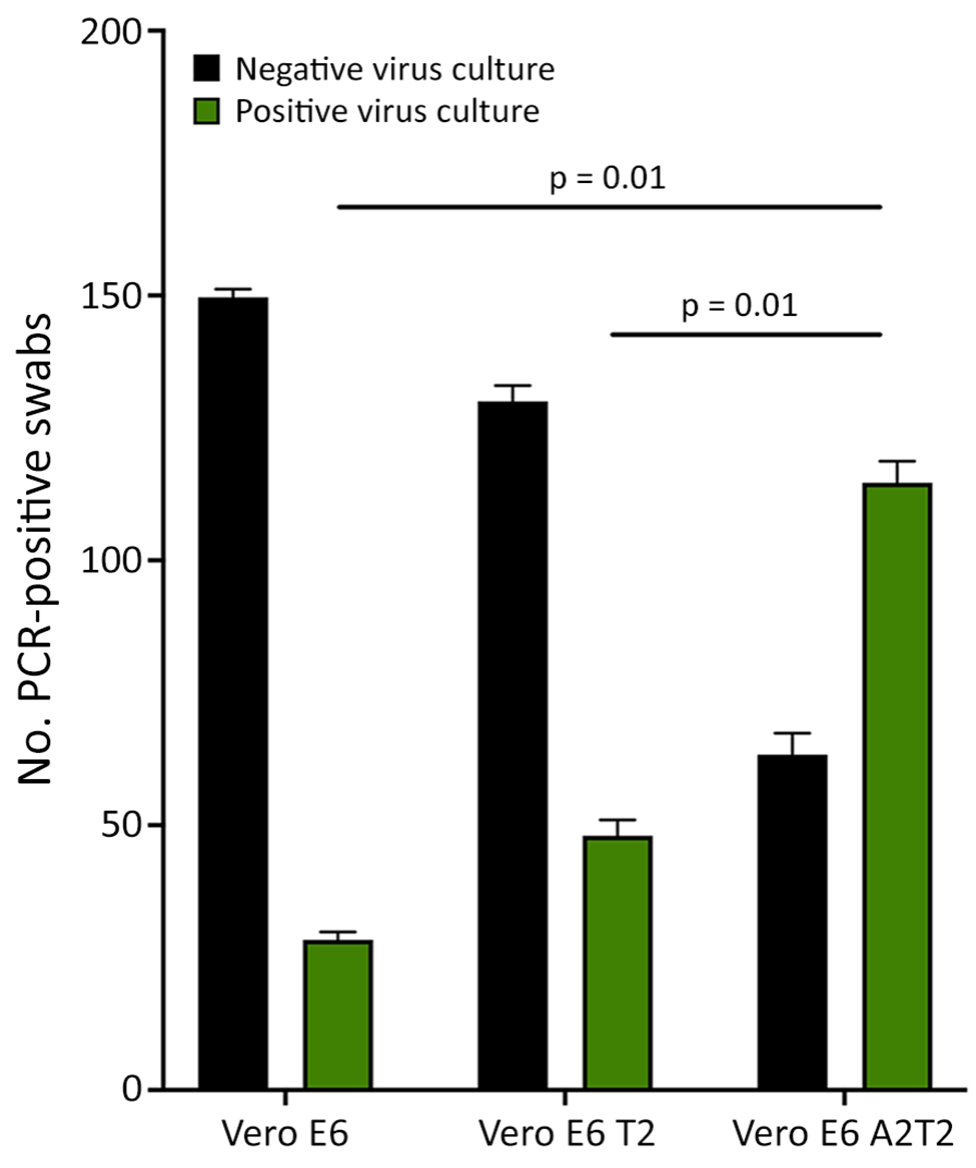
Viral culture isolation rates from nasal swabs of nonimmunocompromised patients infected with SARS-CoV-2 variants of concern from study on the enhanced isolation and detection of COVID-19 in hospitalized patients undergoing antiviral therapy. Nasal swabs (n = 148) PCR-confirmed positive for wild-type, Delta, BA.1, BA.2, BQ, BA.5, and XBB.1 SARS-CoV-2 variants of concern were cultured in triplicate on 3 different cell lines. Error bars indicate SDs. Vero E6 T2, Vero E6 cells expressing transmembrane protease serine 2; Vero E6 A2T2, Vero E6 cells expressing both transmembrane protease serine 2 and angiotensin-converting enzyme 2.

We confirmed nasal VTM samples (n = 148) by qRT-PCR and sequencing to contain several SARS-CoV-2 variants, including wild-type, Delta, BA.1, BA.2, BQ, BA.5, and XBB.1. We inoculated samples in triplicate onto the 3 cell lines to evaluate their capacity for isolating viruses from swab specimens that were positive for variants other than BA.5. The mean +SD isolation success rates were 28.3 +1.24 per 148 samples for Vero E6, 48 +2.44 for Vero E6 T2, and 114.6 +3.29 for Vero E6 A2T2, suggesting greater efficiency of Vero E6 A2T2 cells (p<0.001) (Figure 2).

To assess suitability for high titer virus stock production, we inoculated nasal swab specimens from 3 patients onto all 3 cell lines and ALI primary human nasal epithelial cultures. We subsequently passaged virus isolates in the same cells to obtain second-generation virus stocks. The final log mean +SD viral titers (measured by TCID_50_ assays that used Vero E6 A2T2) after the second passage in ALI cultures were 7.3 +0.1, 7.5 +0.1, and 7.0 +0.3 for the 3 patients. In comparison, second-passage titers were 6.3 +0.2, 6.2 +0.3, and 6.6 +0.3 for Vero E6; 6.1 +0.3, 5.2 + 0.3, and 5.5 +0.2 for Vero E6 T2; and 5.1 +0.2, 5.5 +0.3, and 5.5 +0.2 for Vero E6 A2T2. Thus, although sensitive for viral isolation, Vero E6 A2T2s produced viral stocks with much lower viral titers (Table 3).

**Table 3:** Three clinical specimens confirmed positive by real time reverse transcriptase PCR for the SARS-CoV-2 BA.5 Omicron variant from study on the enhanced isolation and detection of COVID-19 in hospitalized patients undergoing antiviral therapy

Nasal swab ID	ALI	Vero E6	Vero E6 T2	Vero E6 A2T2	p value
1	7.3 ±0.1	6.3 ±0.2	6.1 ±0.3	5.1 ±0.2	<0.001
17	7.5 ±0.1	6.2 ±0.3	5.2 ±0.3	5.5 ±0.3	<0.01
20	7.0 ±0.3	6.6 ±0.3	5.5 ±0.2	5.5 ±0.2	<0.01

*Values are mean ±SD. ID, identification.

**Figure 3:**
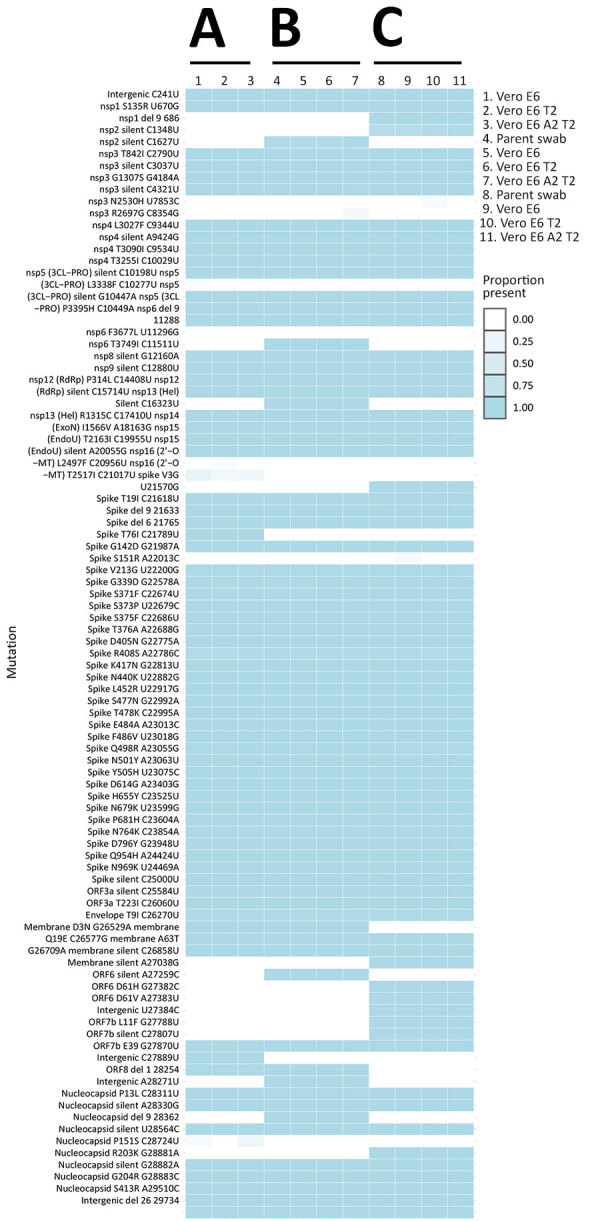
Heatmap of viral whole genome sequences of patient swab specimens after first passage in the 3 cell lines Vero E6, Vero E6 T2, and Vero E6 A2T2 from study on the enhanced isolation and detection of COVID-19 in hospitalized patients undergoing antiviral therapy. Columns represent sequenced samples; rows correspond to mutations relative to the wild-type reference SARS-CoV-2 strain. Darker blue shades indicate an increased prevalence of specific mutations relative to the wild-type strain. Del, deletion; nsp, nonstructural protein; ORF, open reading frame; RdRp, RNA-dependent RNA polymerase; Vero E6 T2, Vero E6 cells expressing transmembrane protease serine 2; Vero E6 A2T2, Vero E6 cells expressing both transmembrane protease serine 2 and angiotensin-converting enzyme 2.

**Figure 4:**
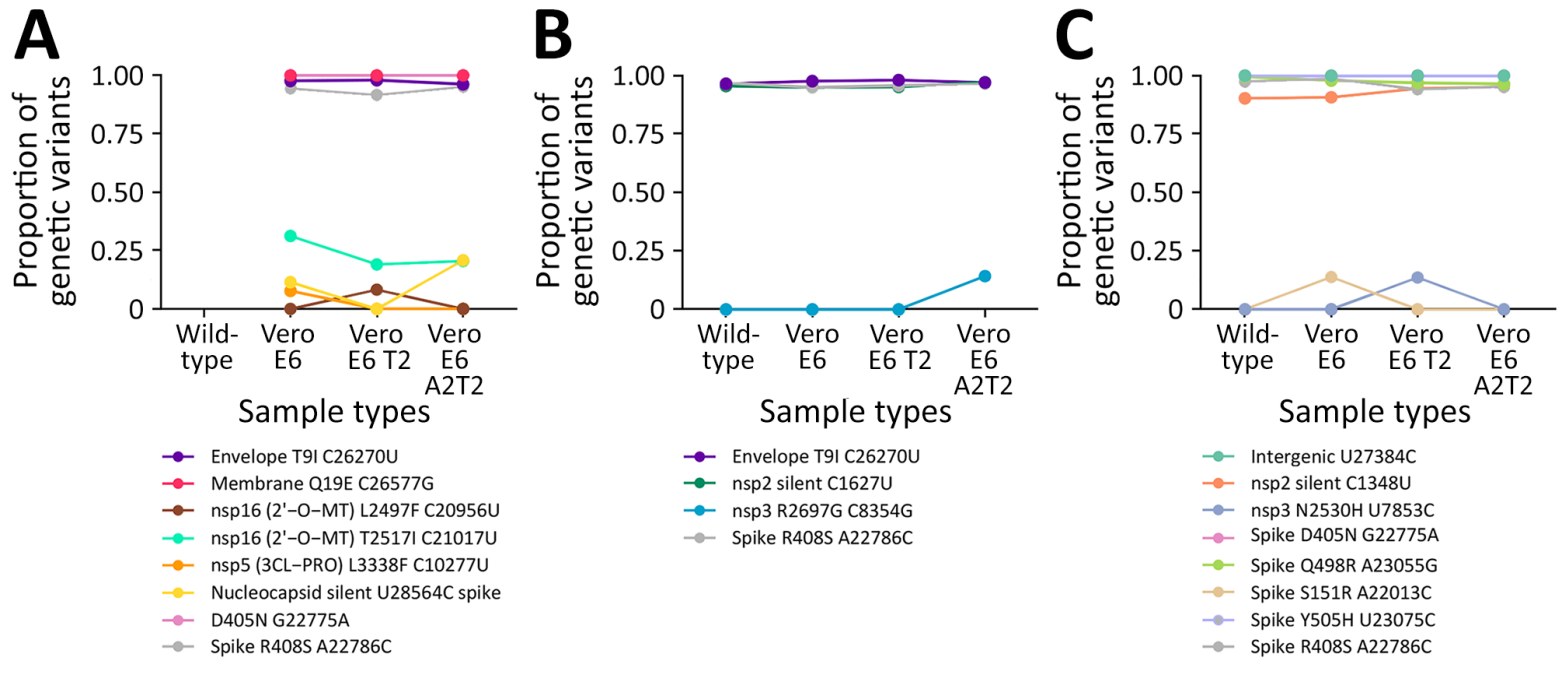
Minor variants identified after passage 1 from SARS-CoV-2 BA.5 Omicron variant positive nasal swab specimens in 3 cell lines Vero E6, Vero E6 T2, and Vero E6 A2T2 from study on the enhanced isolation and detection of COVID-19 in hospitalized patients undergoing antiviral therapy. This figure shows the minor variants observed in the 3 virus culture isolates obtained after passage 1 from nasal swab samples from patients 1, 17, and 20 that were SARS-CoV-2 positive and were a BA.5 Omicron variant. Vero E6 T2, Vero E6 cells expressing transmembrane protease serine 2; Vero E6 A2T2, Vero E6 cells expressing both transmembrane protease serine 2 and angiotensin-converting enzyme 2.

We selected 3 clinical specimens (patients 1, 17, and 20) confirmed by qRT-PCR to be positive for the SARS-CoV-2 BA.5 Omicron variant and each sample was inoculated onto Vero E6, Vero E6 T2, and Vero E6 A2T2 cell lines. We conducted whole-genome sequencing on the parent swab and viral isolates obtained after the first passage in each of the cell lines to assess if there were any cell line-specific mutations introduced during culture. We also whole-genome sequenced the original swab material from patients 17 and 20, but insufficient nucleic acid was available from patient 1 for sequencing. Comparative genomic analysis revealed no noteworthy differences between the viral genomes from the parental swab material and those derived from each cell line (Figure 3). Furthermore, there were no consensus mutations, and the changes in minor variants were fairly limited, with <0.15 change in proportion (Figure 4).

**Figure 5:**
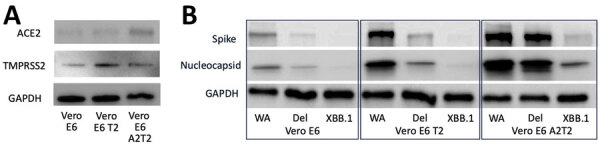
Efficiency and possible routes of infectious viral particle internalization of SARS-CoV-2 in Vero E6, Vero E6 T2, and Vero E6 A2T2 cell lines from study on the enhanced isolation and detection of COVID-19 in hospitalized patients undergoing antiviral therapy. Samples were separated by using sodium dodecyl sulfate–polyacrylamide gel electrophoresis and transferred to a polyvinylidene difluoride membrane for detection of ACE2, TMPRSS2, GAPDH (A) or SARS-CoV-2 spike and nucleocapsid antibodies (B). GAPDH, glyceraldehyde-3-phosphate dehydrogenase; ACE2, angiotensin-converting enzyme 2; del, deletion; TMPRSS2, transmembrane protease serine 2; Vero E6 T2, Vero E6 cells expressing TMPRSS2; Vero E6 A2T2, Vero E6 cells expressing both TMPRSS2 and ACE2.

Successful virus culture isolation depends on the efficient entry of viral particles from clinical specimens into susceptible cells through cell surface or endosomal pathways and then by replication. To compare the efficiency and route of viral entry in the 3 Vero-derived cell lines, we inoculated nasal swab samples qRT-PCR-positive for SARS-CoV-2 variants WA.1, Delta, and XBB.1 (viral titers measured by Vero E6 A2T2) at a MOI of 0.01. Western blot confirmed higher expression levels of TMPRSS2 in Vero E6 T2 cells and ACE2 in Vero E6 A2T2 cells (Figure 5, panel A).

**Figure 6:**
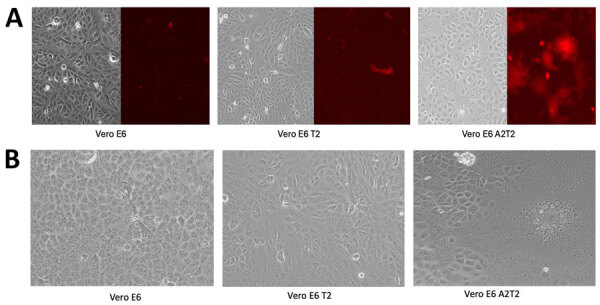
Efficiency and possible routes of infectious viral particle internalization for mCherry-labeled wild-type SARS-CoV-2 virus in Vero E6, Vero E6 T2, and Vero E6 A2T2 cell lines from study on the enhanced isolation and detection of COVID-19 in hospitalized patients undergoing antiviral therapy. A) Inoculated cells, singular cells or fused (in red). B) Cytopathic effects induced by SARS-CoV-2 XBB.1 viral inoculation, focal areas of rounded cells or regions of fused cells forming syncytia or giant cell structures across the different cell lines. Vero E6 T2, Vero E6 cells expressing transmembrane protease serine 2; Vero E6 A2T2, Vero E6 cells expressing both transmembrane protease serine 2 and angiotensin-converting enzyme 2.

We harvested cells 12 hours postinfection to probe for SARS-CoV-2 spike and nucleocapsid proteins. We detected nucleocapsid and spike protein in all the cell lines that were inoculated with nasal swab material positive for wild-type and Delta strain, with the highest expression observed in Vero E6 A2T2. In contrast, we only observed nucleocapsid and spike expression with Vero E6 A2T2 cells inoculated with nasal swab material positive for XBB.1 variant (Figure 5, panel B). Infection with mCherry-expressing wild-type virus at 0.01 MOI revealed much greater membrane fusion events in Vero E6 A2T2 cells, as indicated by broader areas of red fluorescence aligned with cytopathic effect seen from the brightfield image compared with the other 2 cell lines that demonstrated a more localized cellular infection at 12 hours postinfection (Figure 6, panel A). Cytopathic effect observations at 12 hours postinfection for wild-type, Delta, and XBB.1 variants at 0.01 MOI revealed numerous pronounced syncytia and fewer single cell infections in Vero E6 A2T2, whereas Vero E6 and Vero E6 T2 cells primarily exhibited isolated infected cells (Figure 6, panel B).

**Figure 7:**
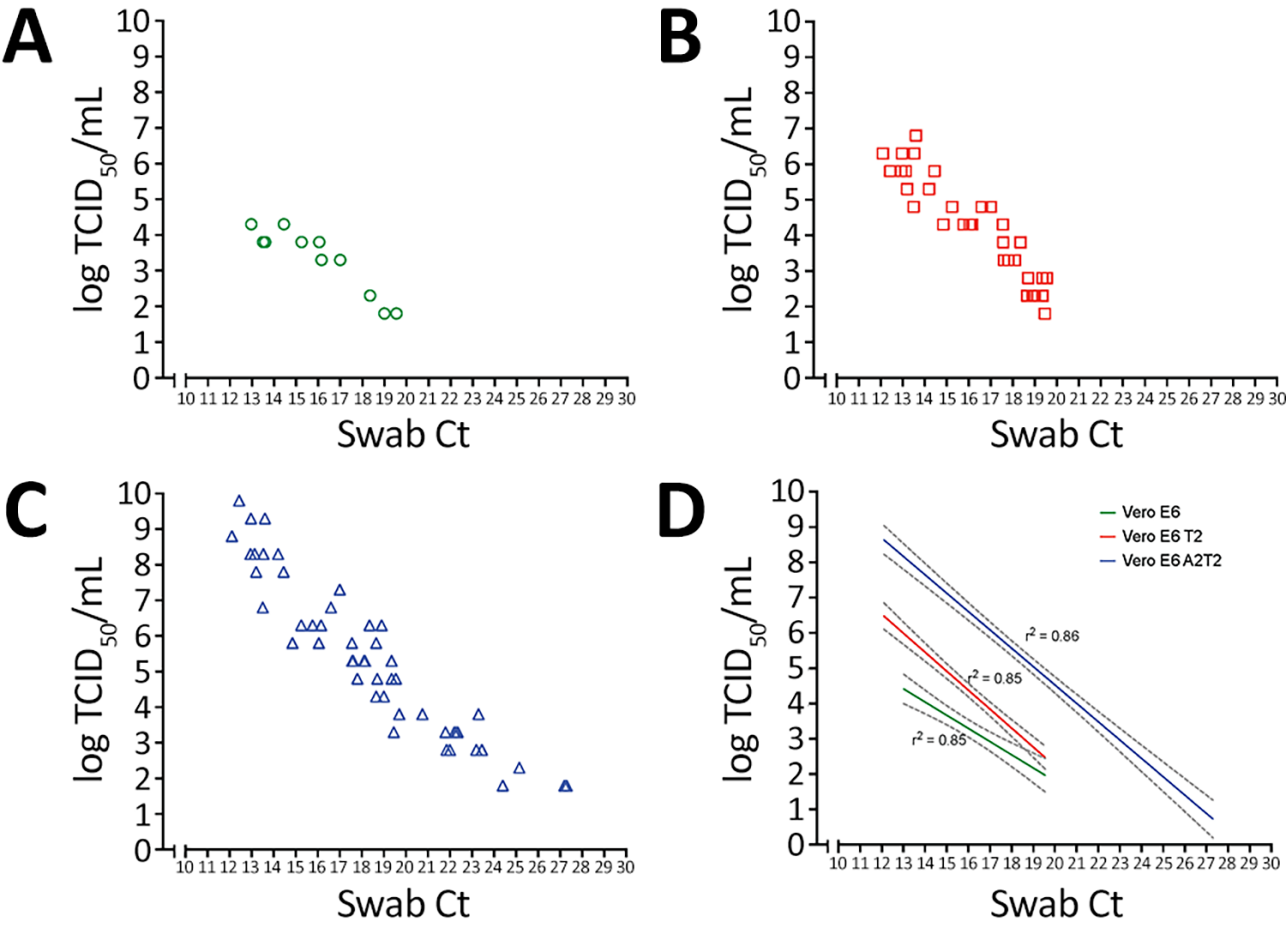
Threshold sensitivity SARS-CoV-2 viral titers by TCID_50_ assays that used different cell types in upper respiratory tract specimens across a range of Ct values from a study on the enhanced isolation and detection of COVID-19 in hospitalized patients undergoing antiviral therapy. A–C) Viral titers were measured in samples with varying Ct values using TCID_50_ assays for 3 cell lines: A) Vero E6, B) Vero E6 T2, and C) Vero E6 A2T2. D) Pearson correlation coefficient of the respiratory viral titers determined by each cell line versus the Ct value. Dotted lines indicate 95% CIs. Ct, cycle threshold; TCID_50_, 50% tissue culture infectious dose; Vero E6 T2, Vero E6 cells expressing transmembrane protease serine 2; Vero E6 A2T2, Vero E6 cells expressing both transmembrane protease serine 2 and angiotensin-converting enzyme 2.

To compare the sensitivity of the 3 Vero E6-derived cell lines in quantifying infectious viral load present in respiratory swab specimens relative to Ct values, we performed a modified TCID_50_ assay on the basis of detecting foci of infection by using clinical samples from a subset of hospitalized patients with SARS-CoV-2 infection. Modified TCID_50_ assays conducted by using parental Vero E6 cells yielded viral titers of log 1.8–4.3 TCID_50_/mL. In contrast, assays that used Vero E6 T2 cells demonstrated higher viral titers of 1.8–6.8 TCID_50_/mL, and assays that used Vero E6 A2T2 cells exhibited the broadest and most sensitive detection range, spanning 1.8–9.8 TCID_50_/mL. Across all 3 cell lines, viral titers demonstrated a strong inverse correlation with the qRT-PCR Ct values of the respective respiratory samples, with inverse Pearson correlation coefficients of 0.85 for both Vero E6 and Vero E6 T2, and 0.86 for Vero E6 A2T2. Of note, Vero E6 A2T2 cells demonstrated superior sensitivity, consistently yielding higher infectious titers and detecting infectious virus even in specimens with the highest Ct values. The highest Ct threshold level for detecting infectious virus was 27.3 (Figure 7).

**Figure 8:**
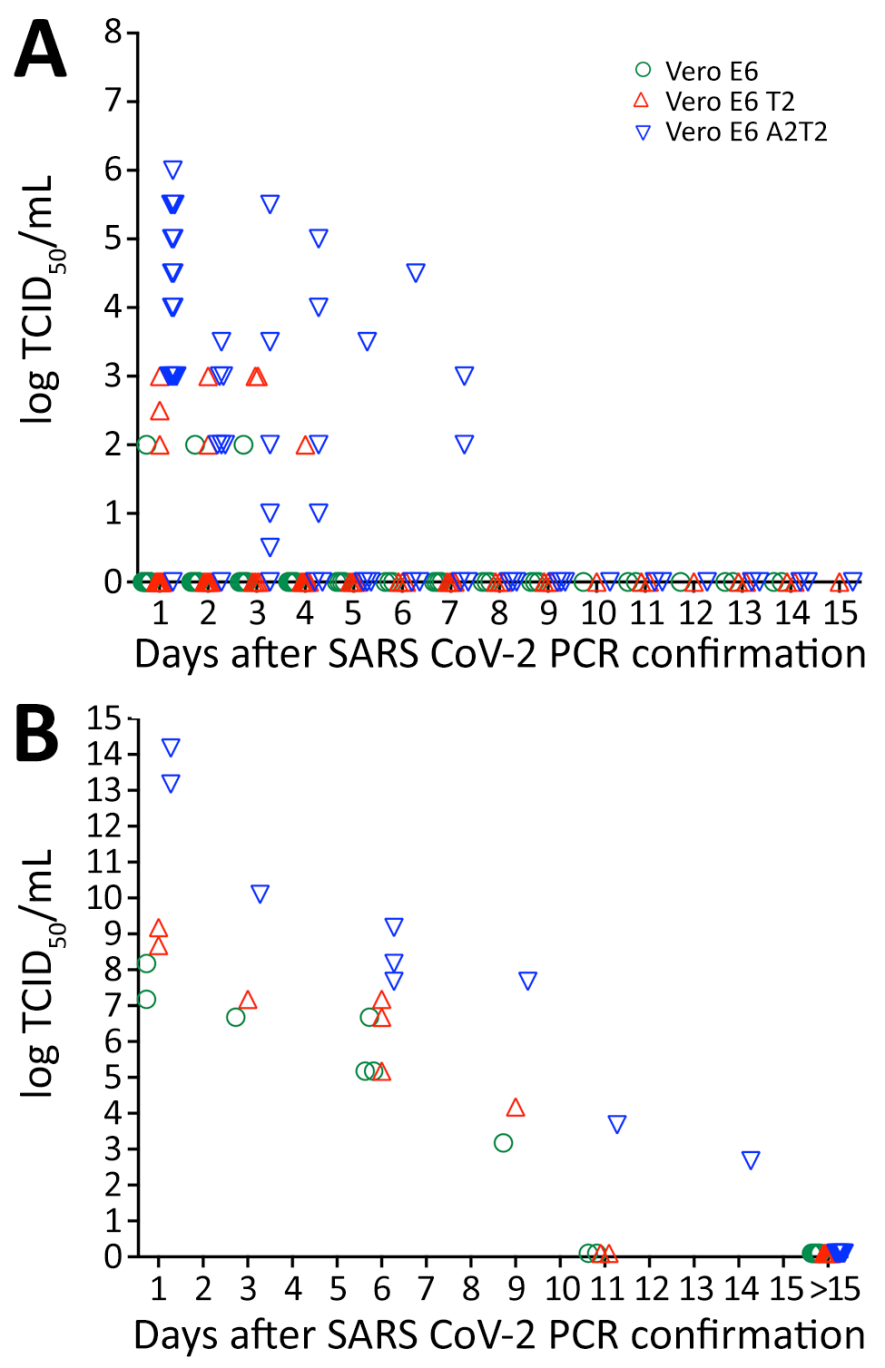
Infectious SARS-CoV-2 virus detection over time in the upper and lower respiratory tract of hospitalized patients when assayed using Vero E6, Vero E6 T2, and Vero E6 A2T2 cell lines from study on enhanced isolation and detection of COVID-19 in hospitalized patients undergoing antiviral therapy. Viral titers shed after PCR-confirmed SARS-CoV-2 infection were measured by TCID_50_ assays that used the 3 cell lines. A) Upper respiratory tract viral titers obtained from nasal swab specimens; B) lower respiratory tract titers obtained from endotracheal aspirates. TCID_50_, 50% tissue culture infectious dose; Vero E6 T2, Vero E6 cells expressing transmembrane protease serine 2; Vero E6 A2T2, Vero E6 cells expressing both transmembrane protease serine 2 and angiotensin-converting enzyme 2.

After assessing the correlation of TCID_50_ infectious titers with Ct values in each cell type in hospitalized patients, we measured the duration of the infectious viral production by the patients after qRT-PCR confirmed SARS-CoV-2 infection (n = 68) by TCID_50_ assays that used Vero E6, Vero E6 T2, and Vero E6 A2T2 cell lines. First, we measured the upper respiratory tract viral titers from a subset of patients (n = 38) (Figure 8, panel A). Those patients received remdesivir, Paxlovid, or monoclonal antibody therapy. The titers obtained in Vero E6 revealed only 3 patients (3/38) to have infectious virus in their upper respiratory tract up to 3 days (log 2 TCID_50_/mL). When Vero E6 T2 cells were used, we detected infectious virus (log 2–3 TCID_50_/mL) in 7 patients (7/38) up to 4 days. However, Vero E6 A2T2 cells detected infectious virus in more patients (16/38), with viral titers of log 0.5–6 TCID_50_/mL, and 2 patients showed detectable virus at 7 days. 

We also assessed lower respiratory tract viral titers in a subset of patients (n = 30) by using endotracheal aspirates. The lower respiratory tract titers of those patients were log 3.17–8.17 TCID_50_/mL from assays that used Vero E6 cells, log 4.17–9.17 TCID_50_/mL from assays that used Vero E6 T2 cells, and log 2.67–14.17 TCID_50_/mL from assays that used Vero E6 A2T2 cells (Figure 8, panel B). Assays that used Vero E6 and Vero E6 T2 cells showed viral production in the lower respiratory tract up to 9 days after PCR confirmation and assays that used Vero E6 A2T2 cells showed infectious virus in the lower respiratory tract up to 11 days after PCR confirmation. Vero E6 A2T2 cells appear to detect infectious virus production from patients for substantially longer than the Vero E6 that is traditionally used for virus isolation.

## Discussion

Through a comparative evaluation of 3 Vero E6–derived cell lines, our data provide insights for improving the detection and quantification of infectious SARS-CoV-2 from clinical samples, including hospitalized, critically ill, or immunocompromised patients. This investigation highlights the substantial value of the optimization of the level of TMPRSS2 and ACE2 expression in the cell lines used for the isolation and quantification of infectious virus from clinical samples. Compared with PCR-based methods or the traditional Vero cells, cell lines expressing both the ACE2 receptor and TMPRSS2 protease were much more effective and had a multifold increase in sensitivity for detecting infectious virus. Although we did not find a similar comprehensive mechanistic comparison in the literature, similar findings have been reported by using different Vero E6 cell lines (*19*–*21*). We observed that Vero E6 A2T2 consistently outperformed the parental Vero E6 and intermediate Vero E6 T2 in virus isolation efficiency, infectious titer quantification, and sensitivity of detection from specimens with low viral RNA loads. This performance was evident in both standard isolation and TCID_50_ assay viral quantification. In contrast, when titers of viral stock grown in the different cells were compared, Vero E6 A2T2 performed poorly, likely because of the rupture of syncytia before optimal titers were reached. This poor performance suggests that although ideal for virus isolation, Vero E6 A2T2 might not be ideal for virus stock preparation. Of note, Vero E6 A2T2 cells did not induce adaptation mutations in the viral genome after initial passages, indicating they preserve virus integrity while enhancing sensitivity and are well-suited for downstream genomic and phenotypic analyses. Mechanistic studies confirmed initiation of early replication after 12 hours of incubation, higher levels of spike protein expression, and robust syncytia formation of fluorescent reporter viruses, reinforcing the utility of Vero E6 A2T2 for detection, isolation, and quantification. This confirmation underscores the value of implementing optimized virus detection methods in the clinical settings, particularly when monitoring viral kinetics in patients with prolonged infections. Of note, although Vero E6 cells expressing only ACE2 might slightly improve over parental cells because of higher receptor density, lacking TMPRSS2 directly affects entry, resulting in inferior efficiency versus TMPRSS2 expressing cell lines (*30*). We found that a Ct value of 27.3 would be the threshold of infectious virus isolation by using the high sensitivity Vero E6 A2T2 line, although this threshold might depend on different variants.

One limitation of this study is that we did not have enough sample size to get estimates for each variant. Accurate measurement of infectious virus is also critical in basic science and animal studies to achieve correct conclusions. Vero E6 A2T2 cells detected higher titers and durations of viral production in patients relative to other cell lines. A second limitation of the study was that the period between patients’ symptom onset and hospital admission after PCR confirmation likely varied among different patients. Therefore, although the relative sensitivity of the different cell lines is clear, the kinetics of infectious virus decline is not generalizable. In our patients, infectious virus was detected up to 11 days post-PCR, and titers reached log 14.17 TCID_50_/mL in endotracheal aspirates. This study highlights the need to apply optimal infectious virus detection methodologies to studies of therapy and infection control to prevent prolonged viral transmission.

In conclusion, the Vero E6 A2T2 cell line represents a sensitive, robust, and reliable platform for SARS-CoV-2 isolation and quantification particularly in complex cases involving immunocompromised patients. Our findings offer an optimized methodological framework for enhanced virologic surveillance and therapeutic monitoring, supporting better clinical and public health management during outbreaks.
